# Mental Health and Substance Use in NCAA Athletes in the Context of the COVID-19 Pandemic and Lockdown

**DOI:** 10.7759/cureus.29836

**Published:** 2022-10-02

**Authors:** Maddison McLellan, Carly Heffernan, Jason Xu, John Billimek, Brian Y Kim

**Affiliations:** 1 Orthopedic Surgery Department, University of California, Irvine, Irvine, USA; 2 Family Medicine, University of California, Irvine, School of Medicine, Irvine, USA; 3 Family Medicine, University of California San Diego Health, San Diego, USA

**Keywords:** covid-19, mental health, substance use, ncaa athlete, covid-19 pandemic

## Abstract

Background

Although the coronavirus disease 2019 (COVID-19) pandemic had pervasive effects on the lives of individuals, its influence on the mental health of collegiate athletes remains unknown. This study aimed to assess changes in mental health and substance use in National Collegiate Athlete Association (NCAA) Division I athletes in Southern California during the COVID-19 pandemic.

Methodology

An online survey was created using the Qualtrics software (Qualtrics, Provo, Utah). NCAA Division I athletes in Southern California completed preseason surveys querying indices of mental health, substance use, and injury in the year before the COVID-19 pandemic (March 2019 to March 2020) and during the pandemic (March 2020 to March 2021). The athletes filled out the survey from June 2021 to September 2021. Participants were asked how likely they were to agree with the following statements: I have felt physically prepared for athletic competitions, I have been satisfied with my mental health, and I have had adequate sleep. Participants were also asked to compare their substance use between the two time periods. Sociodemographic information regarding participants’ age, gender, sports team, as well as year in sport and school was also collected. Group comparison analyses were performed using Fisher’s exact test. Correlations between mental health measures and other variables were examined using Spearman’s correlation coefficients.

Results

A total of 189 athletes completed the survey (out of the 259 surveys that were started). Females were significantly less likely to feel satisfied with mental health (p < 0.01) and physically prepared for sport (p < 0.01). Across all respondents, satisfaction with mental health was positively correlated with adequate sleep (p < 0.01) and physical preparedness for sport (p < 0.01) and negatively correlated with injury (p < 0.05). There was no significant correlation between mental health status and history of COVID-19 infection (p = 0.84). The vast majority of athletes reported no significant change in substance use pre- to post-pandemic, with no differences according to sex.

Conclusions

The COVID-19 pandemic had a differential impact on the mental health of female versus male NCAA athletes. Mental health was correlated with sleep, physical preparedness, and being injury-free but not with a history of COVID-19 infection. Despite reports indicating increased substance use in the general population, athletes in this group reported no change in licit and illicit substance use.

## Introduction

Over the past two years, the coronavirus disease 2019 (COVID-19) pandemic has impacted nearly every facet of life, including work, education, and socialization, generating widespread concerns about mental health. Within weeks of the first reported cases in the United States, 13.6% of adults reported symptoms of serious psychological distress, up from 3.9% the year prior [1]. As the pandemic has worn on, concerning trends in loneliness, social disconnection, and substance use have also emerged [2,3]. These reports reinforce research demonstrating large-scale disasters, whether natural or human-made, are almost always accompanied by increases in depression, posttraumatic stress disorder, substance use, and other mental health and behavioral disorders [4].

Young adults, especially those in the midst of major life transitions, are particularly vulnerable to mood and substance use disorders [5]. Young athletes appear to be at least as susceptible to depression as the general public, often facing a myriad of stressors, including public scrutiny, career uncertainty, overtraining, and injury [6-11]. They also often defer self-care to prioritize athletic and academic obligations, which may exacerbate stress, worry, and vulnerability to mental health disorders [12]. In a report from the National Collegiate Athlete Association (NCAA) published in 2014, nearly one-third of male and one-half of female student-athletes reported being impacted by anxiety, and approximately one-quarter of all respondents reported feeling depressed [12].

Though the NCAA has included topics related to COVID-19 in its ongoing Student-Athlete Well-Being survey [12], few peer-reviewed studies have examined the effects of the pandemic on the mental health of NCAA athletes. There is also a dearth of specific data on changes regarding substance use patterns in NCAA athletes during the pandemic. Our study aimed to compare NCAA student-athletes’ perceptions of their mental health and substance use before and after the onset of the COVID-19 pandemic in March 2020. A secondary aim was to examine relationships between mental health, injury, and history of COVID-19 infection.

## Materials and methods

Participant recruitment

Participants were recruited during institutional pre-participation evaluations held in the summer of 2021, as well as through emails distributed by team athletic trainers. For inclusion, participants had to be 18 years old or older and on the active roster of a varsity-level sport at the time of participation. All athletes were division 1 athletes from a university in southern California. All years of participation in sport were accepted. Incomplete surveys were excluded. Participation was voluntary, and all respondents gave informed consent before completing the anonymous survey. Given the study design involving the virtual collection of anonymous data, the institution deemed this study IRB-exempt.

Data were collected using a Qualtrics survey platform (Qualtrics, Provo, Utah). The survey was initially distributed at the pre-participation physicals for athletes, and athlete trainers for each team sent one subsequent email approximately two weeks after the last pre-participation physical event. No control group was used for this study as only division 1 collegiate athletes from the University of California, Irvine were queried. Exclusion criteria included only partial completion of the survey. For all inquiries into the effects of the COVID-19 pandemic, participants were asked to compare the period from March 2020-March 2021 (post-pandemic) to March 2019-March 2020 (pre-pandemic). The survey also included demographic information such as age, gender, sports team, year in school, and year in the sport, as well as questions related to mental health, specifically gauging physical preparedness for sport, willingness to access medical care, satisfaction with sleep, satisfaction with mental health, and substance use on a five-point Likert scale. Participants were asked to self-report their history of laboratory-diagnosed COVID-19 infection. The validity of the survey questions was not assessed prior to distribution.

Data analysis

Group comparisons were performed using Fisher’s exact test. Correlations between mental health measures and athletes’ pandemic experiences were examined with nonparametric Spearman’s correlation coefficients.

## Results

The study questionnaire was accessed 259 times and 189 responses were submitted, for a completion rate of 73%. Demographic data are presented in Table 1. Respondents were evenly distributed across gender and year in school. In total, 16 teams were represented in our study, with women’s cross country having the greatest (26 responses, 13.8%) and women’s soccer the fewest (three responses, 1.6%) number of responses. A total of 32 (17%) participants reported a history of laboratory-diagnosed COVID-19 infection.

**Table 1:** Demographic information.

Sport team representation sport	N (% of total)	
Men’s baseball	25 (13.2%)	
Men’s basketball	6 (3.2%)	
Men’s cross country	10 (5.3%)	
Men’s golf	5 (2.6%)	
Men’s soccer	6 (3.2%)	
Men’s tennis	6 (3.2%)	
Men’s track and field	12 (6.3%)	
Men’s volleyball	6 (3.2%)	
Men’s water polo	16 (8.5%)	
Women’s basketball	13 (6.9%)	
Women’s cross country	26 (13.8%)	
Women’s golf	5 (2.6%)	
Women’s soccer	3 (1.6%)	
Women’s tennis	8 (4.2%)	
Women’s track and field	22 (11.6%)	
Women’s volleyball	4 (2.1%)	
Women’s water polo	16 (8.5%)	
Total	189 (100%)	
Demographics		
Gender		
Female	97 (51.3%)	
Male	92 (48.7%)	
Year		
Freshmen	43 (22.8%)	
Sophomore	55 (29.1%)	
Junior	39 (20.6%)	
Senior, fifth year or graduate student	52 (27.5%)	
Years participated in sport		
0	46 (24.3%)	
1	55 (29.1%)	
2	36 (19.0%)	
3	35 (18.5%)	
4	17 (9.0%)	
Redshirt year		
Yes	11 (5.8%)	
No	132 (69.8%)	
Unknown	46 (24.3%)	
History of COVID-19 infection		
Yes	32 (16.9%)	
No	114 (60.3%)	
Unknown	43 (22.7%)	

**Figure 1:**
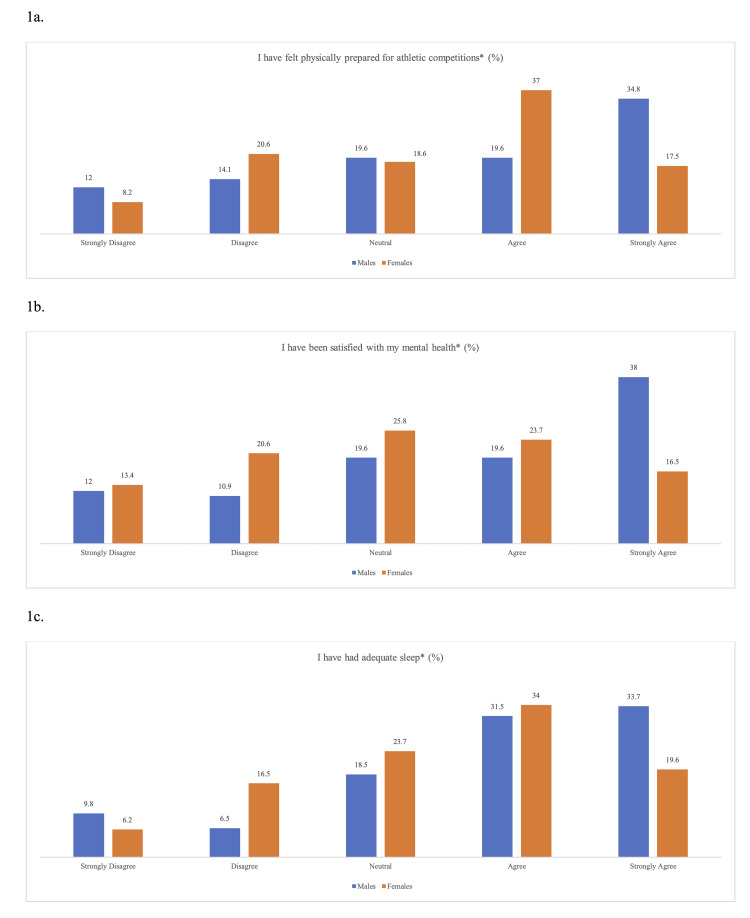
NCAA athlete mental health responses during the COVID-19 pandemic year. (a) Athletes’ response to feeling physically prepared for sport in the COVID-19 pandemic year (March 2020-March 2021). Females were significantly less likely to strongly agree with this statement (*p = 0.022). (b) Athletes’ response to feeling satisfied with mental health in the COVID-19 pandemic year (March 2020-March 2021). Females were significantly less likely to strongly agree with this statement (*p = 0.015). (c) Athletes’ response to having adequate sleep in the COVID-19 pandemic year (March 2020-March 2021). No significant difference in response between genders (*p = 0.59).

When asked about the post-pandemic period (March 2020-March 2021), females were significantly less likely than males to strongly agree with the following statements: “I have felt physically prepared for athletic competitions” (17.5% of women and 34.8% of men, p = 0.022) and “I have been satisfied with my mental health” (16.5% of women and 38.0% of men, p = 0.015). There was also a non-significant trend toward females reporting less adequate sleep than males (19.6% of women and 33.7% of men, p = 0.059) (Figure 1).

Across all respondents, satisfaction with mental health was positively correlated with adequate sleep (rs = 0.651, p < 0.01) and feeling physically prepared for sport (rs = 0.515, p < 0.01), and negatively correlated with injury in the post-pandemic year (athletes who sustained fewer injuries were more likely to be satisfied with their mental health, rs = -0.153, p < 0.05). Of note, there was no significant correlation between history of COVID-19 infection and mental health status (rs = 0.012, p > 0.05), adequate sleep (rs = 0.086, p > 0.05), or physical preparedness for sport (rs = 0.022, p > 0.05). There was also no correlation between COVID-19 infection and injury during the post-pandemic period (rs = 0.013, p > 0.05).

**Figure 2:**
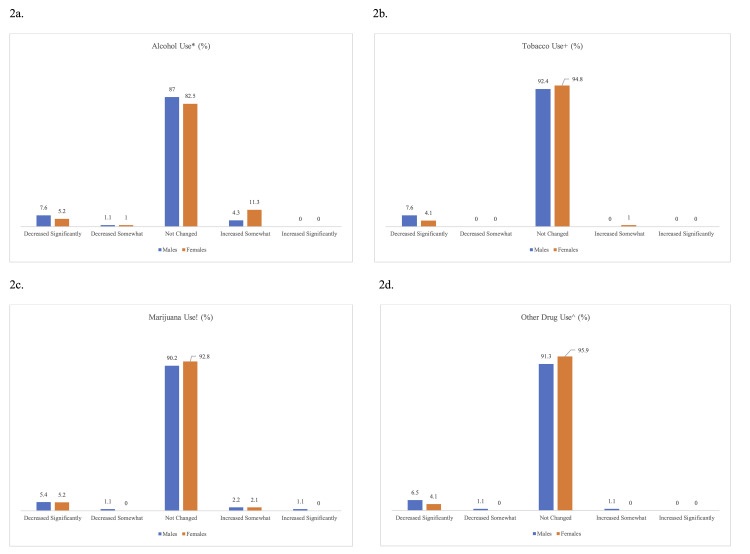
Substance use among NCAA athletes during the COVID-19 pandemic. (a-d) Alcohol, tobacco, marijuana, and other drug use during the COVID-19 pandemic year (March 2020-March 2021). No difference in response between genders for any substances (alcohol *p = 0.25, tobacco +p = 0.37, marijuana !p = 0.93, other drugs ^p = 0.38).

Data on changes in substance use is presented in Figure 2. Participants were only queried about changes in substance use rather than their baseline substance use habits. The vast majority of respondents reported no significant change in consumption of any substances pre- versus post-pandemic, and there was no significant difference in reported use between females and males. In all cases, a substantially greater percentage of respondents reported a significant decrease in use versus a significant increase in use. There was no significant correlation between a history of COVID-19 infection and changes in substance use (rs = 0.008 for alcohol, rs = -0.052 for tobacco, rs = -0.009 for marijuana, and rs = -0.001 for other drugs, all p > 0.05).

## Discussion

Our study highlights aspects of the mental health experience during the COVID-19 pandemic, adding to our understanding of how NCAA student-athletes respond to crises. In our analysis, the impact of COVID-19 on athletes’ mental health showed variance by gender, aligning with research conducted prior to the pandemic [12].

Rocha and Osorio have shown that female athletes, as well as younger athletes and athletes with less experience in sports, are more susceptible to competitive anxiety than their male counterparts [8]. Similar findings have been reported in elite female athletes who are more likely to be diagnosed with mental health problems and experience more associated adverse life events than males [13]. This finding of female sex as a risk factor for mental health issues in the NCAA Division I athlete population has been mirrored in the general population during the pandemic [2,3], corroborating previous research showing that athletes’ susceptibility to mental health is similar to the larger population [13,14]. Additional research is needed to clarify the factors that contribute to the differential response of female student athletes’ mental health to crises like the COVID-19 pandemic.

We also found that the COVID-19 pandemic did not largely alter the substance use patterns of our study population. This was unexpected in light of reports of increased substance use, particularly alcohol, in the general adult population during the pandemic [15,16]. One hypothesis is that sports participation may have offered a protective effect against an increase in substance use during this time of crisis. This is supported by a large body of pre-pandemic research suggesting sport has a protective effect against alcohol, tobacco, and marijuana use [17-19].

However, the factors leading to changes in substance use among collegiate athletes are likely multifaceted. A recent study of college students by Graupensperger and colleagues showed that social stressors during the pandemic were associated with increased alcohol use, whereas illness and school-related stressors were associated with less alcohol use [20]. Additionally, due to widespread lockdown measures and temporary cessation of in-person classes, many student-athletes moved back home during the pandemic, potentially distancing them from on- or off-campus settings, such as parties, that may have ordinarily presented an increased opportunity for substance use. Lastly, though our study did not include non-athletes, future research may help clarify whether the stressors faced by collegiate athletes during the pandemic mirrored those in the general collegiate population.

Limitations

Our study relied on participant self-report that is vulnerable to response and recall bias. We also had a relatively small sample size, with student-athletes from a single institution, which may limit the generalizability of our findings. Additionally, a high proportion of our female respondents (50%) were track and field or cross country athletes, which is another potential source of bias. Because there was no control group of non-athletes, it is not known how our study population’s experiences differed from that of the general college population. Additionally, we did not assess the validity of our survey instrument and did not use validated survey instruments (i.e., Patient Health Questionnaire-9).

## Conclusions

The impact of the COVID-19 pandemic on indices of NCAA athlete mental health is multifactorial and may differ by gender, with females showing an increased susceptibility. Moreover, despite reports indicating increased substance use in the general population during the COVID-19 pandemic (particularly alcohol), the vast majority of athletes in our study reported no change in licit or illicit substance use. Further research should explore mechanisms underpinning variations in mental health response to provide tailored support to athletes in times of crisis.
